# Intra-urban microclimate investigation in urban heat island through a novel mobile monitoring system

**DOI:** 10.1038/s41598-021-88344-y

**Published:** 2021-05-06

**Authors:** Ioannis Kousis, Ilaria Pigliautile, Anna Laura Pisello

**Affiliations:** 1 CIRIAF - Interuniversity Research Center, University of Perugia, Via G. Duranti 67, 06125 Perugia, Italy; 2 Department of Engineering, University of Perugia, Via G. Duranti 97, 06125 Perugia, Italy

**Keywords:** Energy infrastructure, Environmental sciences, Environmental social sciences, Energy science and technology, Engineering

## Abstract

Monitoring microclimate variables within cities with high accuracy is an ongoing challenge for a better urban resilience to climate change. Assessing the intra-urban characteristics of a city is of vital importance for ensuring fine living standards for citizens. Here, a novel mobile microclimate station is applied for monitoring the main microclimatic variables regulating urban and intra-urban environment, as well as directionally monitoring shortwave radiation and illuminance and hence systematically map for the first time the effect of urban surfaces and anthropogenic heat. We performed day-time and night-time monitoring campaigns within a historical city in Italy, characterized by substantial urban structure differentiations. We found significant intra-urban variations concerning variables such as air temperature and shortwave radiation. Moreover, the proposed experimental framework may capture, for the very first time, significant directional variations with respect to shortwave radiation and illuminance across the city at microclimate scale. The presented mobile station represents therefore the key missing piece for exhaustively identifying urban environmental quality, anthropogenic actions, and data driven modelling toward risk and resilience planning. It can be therefore used in combination with satellite data, stable weather station or other mobile stations, e.g. wearable sensing techniques, through a citizens’ science approach in smart, livable, and sustainable cities in the near future.

## Introduction

Within recent decades the rural-to-urban population flow has substantially increased. In 2016, 54% of the world population was reported to live in urbanised areas. At the same time, future projections of urbanization rates are rather alarming. It is expected that by 2050 and 2100 the corresponding fraction will increase up to 66% and 85% respectively^1^. Urbanization is typically followed by high population and building density and consequent land-use and surface alterations, e.g. deforestation, loss of farmland^2,3^. Natural-to-urban land alterations affect in turn the local energy balance of cities and thus their microclimatic characteristics and thermal environment in particular^4,5^. As a result, cities tend to systematically experience higher surface and air temperatures as compared to the surrounding rural areas, a phenomenon reported as Urban Heat Island (UHI) effect^6–9^. The driving physics behind UHI is the reduction in latent heat flux and increase in sensible heat flux^10,11^. UHI is a significant human-induced environmental change that poses threats to human life. For instance, increased morbidity and mortality^12^, indoor/outdoor discomfort^13^, air pollution^14,15^, increased energy consumption^16^ and greenhouse gas emissions^17,18^, impaired air and water quality^19^ and intensification of energy poverty on vulnerable social groups during the hot months of the year^20,21^ are just some of UHI consequences that usually are interconnected. Also, UHI is associated with global warming and moreover has been found to synergistically act with heatwaves and amplify their impacts^22–24^. Considering the projections linked to the ongoing climate change, the livability of cities will be seriously endangered^25^. In fact, according to IPCC’s Representative Concentration Pathway (RCP) 8.5, global warming is expected to reach up to 1.5° above pre-industrial levels by 2050, and up to 2.0°–4.9° by 2100 as compared to 1861–1880^26,27^. Thus, heat-related risk within urban canopy layers is likely to increase even more in the very near future, making the urban population particularly vulnerable during periods of hot weather.

Measures for counterbalancing UHI and its aftermaths are deemed of critical importance. In fact, techniques for controlling the variables regulating the urban microclimate are receiving increased attention from academics, urban planners and policy-makers^28–32^. Quantifying, however, the magnitude of each microclimatic parameter is not trivial, especially because affected by dynamic and granular anthropogenic forcing. Instead, due to the complex morphology of urban areas, microclimatic conditions have been found to significantly vary not only among different cities but also among different locations of the very same city^33^. For instance, UHI incidences has been found not only between urban and rural areas but also between urban and suburban areas^34,35^. In general, the profile of each investigated urban microclimate is determined by the unique characteristics of the corresponding area^36^. Therefore, the intrinsic inhomogeneity of urban microclimate needs to be in-depth investigated with respect to the spatio-temporal variations originated from the local morphology, anthropogenic actions, urban planning, and temporal weather conditions^37–39^. For precisely determining the gradient and the intra-urban deviations of microclimatic variables, their spatial extent needs to be thoroughly delineated. Mapping out each variable’s footprint can result in a better understanding and evaluation of cities’ function, as well as decreased biases concerning local phenomena, such as UHI magnitude and its consequent heat stress and risk mapping. Furthermore, more efficient comparison analysis among relevant studies will be feasible^40^.

Traditionally, in-situ meteorological stations have been implemented for measuring parameters such as air temperature and humidity, in and out of the city. For instance, Santamouris et al.^41^ utilised and retrieved data from a network of 23 experimental weather station within the city of Athens and gauged the corresponding UHI magnitude while the same did Yang et al.^16^ in the city of Nanjing, China and Foisard et al.^42^ within the city of Rennes, France by implementing networks of 15 and 22 weather stations, respectively. Similarly, Richard et al.^43^ employed an extended network of 47 fixed air temperature sensors for identifying thermal zones within the city of Dijon, France during a 3-week heatwave. Another sensor network of high density is established by the Birmingham Urban Climate Laboratory and comprises 29 sensors distributed within the entire city of Birmingham^44^. Results of such studies are of critical importance since not only gauge the magnitude of local phenomena, such as UHI, but also shed light on the corresponding mechanisms of urban climate and hence help towards efficient countermeasures. However, since in most cases meteorological stations are sparsely distributed, data retrieved from this method represent a point-wise momentum of each microclimatic variable and not the overall footprint and the corresponding spatial patterns^16^.

To overcome this limitation, recent studies employed remote sensing techniques. In fact, land surface temperature (LST) data from satellites have been widely utilised for measuring the magnitude of microclimatic variables determining surface UHI mainly due to their high spatial resolution. For example, several studies used MODIS LST data^45,46^ for assessing UHI and its drivers within high populated cities in China. However, due to their typically low temporal resolution, together with lack of direct air temperature profiles, data retrieved from satellites cannot be used for evaluating an extensive intra-urban distribution of UHI.

Under this framework, mobile meteorological units, placed typically on motorized vehicles, are becoming popular among academics for determining the spatial variability of microclimatic variables within a city. Table 1 gives a brief overview of relevant published scientific works. Unlike fixed units, mobile stations can offer data acquisition of higher spatial resolution within the desired urban context and thus can be used for identifying the intra-urban diversifications of the parameters affecting the urban microclimate and consequently human well-being. For instance, Hart and Sailor^47^ utilised vehicular temperature traverses in order to determine the spatial variability of air temperature at two-meter height across the metropolitan area of Portland, US. Santamouris et al.^48^ developed a mobile weather station on a telescopic mast placed atop of a vehicular van capable of measuring air temperature, wind speed, and direction at different heights with time-step of 30 s and performed a monitoring campaign before and after the implementation of cool pavements in an urban park at the city of Athens, Greece. Similarly, Busato et al.^34^ assessed UHI incidences within the city of Padua, Italy through the development and utilisation of a mobile weather station built on a vehicle and capable to measure air temperature, relative humidity, and solar global radiation with a time-step of 5 s. Mobile weather units were employed also in the study of Parece et al.^49^ aiming to capture spatial patterns of air temperature (2 s time-step) across the Roanoke, Virginia, USA. Santamouris et al.^50^ also developed a mobile weather station, called “EnergyBus”, that measures air temperature, relative humidity, pressure, and wind speed.

**Table 1:** Studies with mobile traverse monitoring methods. $\hbox {T}_{\text {air}}$ is air temperature, RH is relative humidity, WS and WD are wind speed and direction, SR and LR are short-wave and long-wave incident radiation, P is pressure, E$_v$ is illuminance, $\lambda$ is Longitude, $\phi$ is latitude, h is altitude and dr is precipitation.

Study	Year	Type	Variables	Speed	City	Scale	Access
^51^	1998	Automobile,bicycle	$\hbox {T}_{\text {air}}$	–	Vancouver, CASacramento, US	Macro,micro	Roadways,pedestrians
^52^	2000	Automobile	$\hbox {T}_{\text {air}}$	–	Regina, CA	Macro	Roadways
^47^	2009	Automobile	$\hbox {T}_{\text {air}}$	36 km/h	Portland, US	Macro	Roadways
^48^	2012	Automobile	$\hbox {T}_{\text {air}}$, WS, WD	–	Athens, GR	Macro	Roadways
^34^	2014	Automobile	$\hbox {T}_{\text {air}}$, RHWS, WD	–	Padua, IT	Macro	Roadways
^53^	2014	Bicycle	$\hbox {T}_{\text {air}}$, RH, WSSR radiation	–	Vienna, AT	Micro	Roadways
^54^	2016	Automobile	$\hbox {T}_{\text {air}}$, $\lambda$, $\phi$, h	–	Doha, QA	Macro	Roadways
^49^	2016	Automobile	$\hbox {T}_{\text {air}}$	–	Roanoke, US	Macro	Roadways
^55^	2017	Automobile	$\hbox {T}_{\text {air}}$, RH	50 km/h	Adelaide, AU	Macro	Roadways
^56^	2018	Automobile	$\hbox {T}_{\text {air}}$, $\lambda$, $\phi$, h	–	Los Angeles, US	Macro	Roadways
^37^	2018	Helmet	$\hbox {T}_{\text {air}}$, RH, Pr, SR, E$_v$, WS,WD, $\lambda$, $\phi$, h, $\hbox {CO}_2$, CO, VOC	Walking speed	Gubbio, IT	Micro	Pedestrians
^57^	2019	Automobile	WS, WD $\lambda$, $\phi$, h,dr, P, $\hbox {T}_{\text {air}}$/RH $\hbox {T}_{\text {sur}}$SW, LW	30–40 km/h	Seoul, KR	Macro	Roadways
^58^	2019	Motor vehicle	$\hbox {T}_{\text {air}}$, RH	–	Tainan, TW	Macro	Roadways
^59^	2019	Automobile	$\hbox {T}_{\text {air}}$, RH, WS, SR	18–36 km/h	Delhi,INDhaka, BDFaisalabad, PK	Macro	Roadways
^60^	2019	Wearable	$\hbox {T}_{\text {air}}$, RH	Walking speed	Lyon, FR	Micro	Pedestrians
^50^	2020	Automobile,hexacopter	$\hbox {T}_{\text {air}}$, WSSR, Pr	–	Sydney, AU	Macro	Roadways
^61^	2020	Bicycle	$\hbox {T}_{\text {air}}$, $\lambda$, $\phi$, h	15 km/h	Seville, SP	Macro	Roadways

Moreover, mobile weather stations have been also developed on human-wearable helmet^37^ and trolleys^62^ in order to monitor pedestrian pathways and the corresponding thermal comfort. Nevertheless, areas of vehicular traffic dominate typically the outdoor urban environment^63^. Hence understanding the differing and localized patterns of the parameters that regulate the corresponding microclimate through an exhaustive monitoring techniques is of primary importance. That said, both weather variables, such as temperature, humidity, wind speed and direction, and parameters, such as materials implemented into the built environment, must be taken into consideration. Subsequently, thermal environment and comfort can be efficiently accessed and evaluated for safeguarding the well-being of the citizens.

Under this framework, this study aims to contribute towards detailed monitoring techniques that can identify the environmental quality of urban areas and hence safeguard fine standards of the corresponding risk and resilience planning. It presents the application of an advanced mobile weather station within a city of central Italy. The mobile station can monitor profiles of the main parameters that regulate the lower levels of a typical urban canopy layer. More specifically, it can monitor not only scalar or vector variables such as air temperature, humidity, wind profile, and air pollutants’ concentration, but also directionally dependent variables, such as reflected and diffused shortwave radiation and illuminance that are typically affected by the properties of urban surfaces. The start-up of the novel methodology is demonstrated through two transect campaigns during the winter period of 2020 within the historical city of Perugia, Italy. The main variables that define the quality of a typical urban environment were subsequently mapped and evaluated in terms of intra-urban variations within districts of different morphology.

## Methods

**Figure 1:**
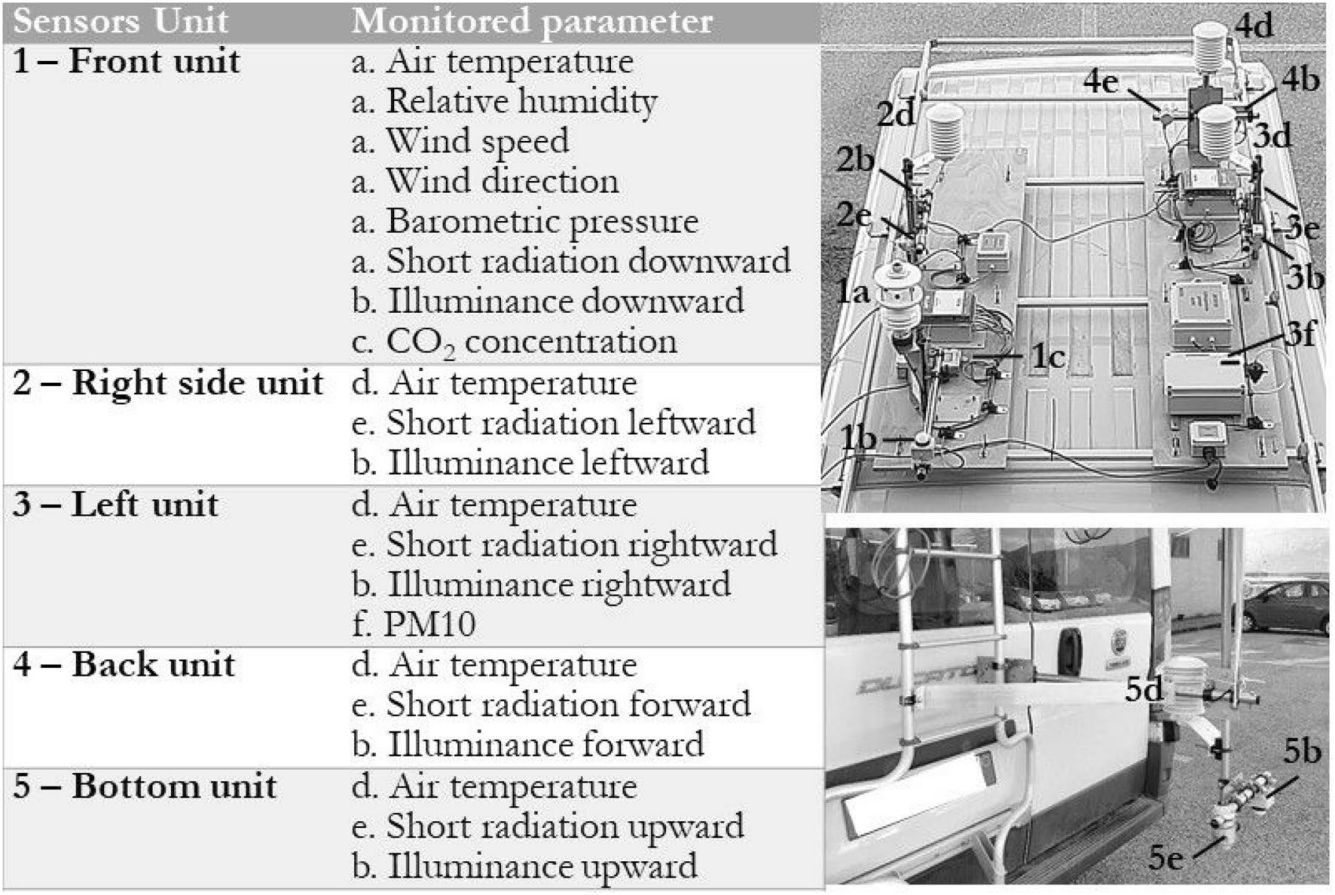
Monitoring system scheme.

The mobile monitoring station includes five units equipped on the 3D surface of the vehicle (Fig. 1). All units are placed above a specifically designed wooden base for minimizing possible affections originated from the van surface. Each unit comprises various sensors (Table 2). The variables measured by the mobile station are air temperature, relative humidity, solar global radiation, illuminance, $\hbox {CO}_2$ and PM10 concentration, and wind speed and direction. Incoming short-wave radiation is among the main regulators of urban microclimate and it is typically measured by a pyranometer facing directly the sky. However, this method is not adequate for accurately depicting solar radiation at a specific height since it compromises shortwave radiation reflected from surfaces at lower heights.

For that reason, in order to ensure an accurate point-wise microclimatic representation of the investigated route, the mobile station presented here is equipped with five pyranometers each one facing towards a different direction. Similarly, five luxmeters were placed towards different orientations for capturing directional illuminance. In more detail, solar global radiation and illuminance are measured each by five different sensors oriented towards (1) the sky, (2) the street, the (3) right, the (4) left, and the (5) backside of the vehicle. The sensors are positioned on the top and back facades of the vehicle so as to minimize both external interferences, e.g. shading effects, and overlapping incidences. Furthermore, in order to ensure an accurate air temperature profile, a corresponding probe is placed in each of the five units. It should be mentioned that in the present study the air temperature probe of unit no. 5 was not utilized. All measurements are taken at the same timestamp every 10 s.

The sensor’s main characteristics, such as operation range, accuracy, and sensitivity error are opted for ensuring the desired level of precision of the corresponding variables. All the main technical characteristics of the installed sensors are summarized in Table 2. Furthermore, since the station is mobile, apart from monitoring micrometeorological parameters, data related to the specific position of the sensors are retrieved by a Global Positioning System (GPS) antenna which is also installed on the vehicle. Therefore, all variable measurements can be directly linked with the corresponding latitude, longitude, and altitude as collected by the GPS antenna that has a Circular Error Probability (CEP) of less than 2.5 m in sky clear view conditions. The spatial accuracy is expected to be lower when the system is located in an urbanized environment, but the error is assumed as acceptable since data points are collected almost every 50 m and the analysis is focused on areas of monitoring path length of 1.2 km at the minimum, as better specified in section 2.1 (Table 4). Furthermore, the GPS antenna is specifically integrated within the wind speed/direction sensor for correcting direction misalignment due to the vehicle’s motion. Further information about the correction algorithm can be found in^64^.

Once the transect is concluded, data loggers of the designed configuration automatically generate a “.csv” file in which all data are saved. Data retrieved from the GPS antenna are also included in the same file and were utilized for intra-urban illustration. In order to minimize overheating incidences and ensure aspiration by vehicle’s motion to the extent possible, the sensors of temperature were placed and ventilated in a PVC radiation shield. All sensors utilised in this study are commercially available products, thus are tested, validated and certified in terms of accuracy by the producing company following the corresponding protocol and standards.

**Table 2:** Characteristics of the sensors comprised by the station.

Sensor	Unit	Monitored variable	Specifications	Orientation
GMX501	1	$\hbox {T}_{\text {air}}$	Accuracy : ±0.3° @ 20° resolution: 0.1°	–
		RH	Accuracy : ±2% @ 20° (10–60% RH) resolution: 1%	–
		WS	Accuracy : ±3% @ 40 m/s resolution: 0.001 m/s	–
		WD	Accuracy : ±3° @ 40 m/s resolution: 1°	
		P	Accuracy : ±0.5 hPa @ 25° resolution: 0.1 hPa	–
		SR	Spectral range: 300–3000 nm 1 W/mq	Downward
DH2021T 8.1	1, 2, 3, 4, 5	E$_v$	Range: 0–10000 lx	Downward, leftward, rightward, forward, upward
EE820	1	$\hbox {CO}_2$	Range: 0–2000 ppm accuracy: ± (50 ppm +2% of measured value)	–
PT100	2, 3, 4	$\hbox {T}_{\text {air}}$	Resolution: 0.1°	–
SR05	2, 3, 4, 5	SR	Spectral range: 285–3000 nm calibration uncertainty: < 1.8%	Leftward, rightward, forward upward
LCT-12	3	PM10	Resolution: 1/4096 Accuracy: < 1%	–

### Monitoring campaigns

Apart from the development and presentation of the mobile monitoring station this study aims to report on two startup monitoring campaigns, performed on weekdays. In order to demonstrate the suitability of the proposed monitoring architecture both campaigns represent data collection at two significant day-times for microclimate investigation purposes, i.e. day-time (around solar noon) and night-time (after sunset and thus in absence of incoming shortwave radiation). The monitoring campaigns were carried out by the authors during the months of January and February of 2020, i.e. in winter conditions. The winter period is a rather under-reported period in terms of mobile microclimate monitoring, while the UHI phenomenon could lead up to +9° in core cities with respect to rural surroundings^65^. Moreover, during the winter period, the city of Perugia is characterized by standard anthropogenic actions, e.g. standard working and school schedules, affecting the thermal environment and air quality, whilst during the summer period, possible biases may occur due to tourism forcing and varied school and working schedules. Previous studies showed that correlations between microclimate parameters and urban morphology are more accurate in terms of statistical significance during clear sky conditions and generally stable boundary conditions^37^. Hence, one clear sky day-time and one clear sky night-time days were chosen for carrying out the presented monitoring campaigns (Table 3).

**Table 3:** Monitoring days and their abbreviation.

Monitoring day	Time of the day	Start-time	End-time	Abbreviation
23/01/2020	Day-time	12:34	13:27	day 1
13/02/2020	Night-time	17:49	19:02	day 2

**Figure 2:**
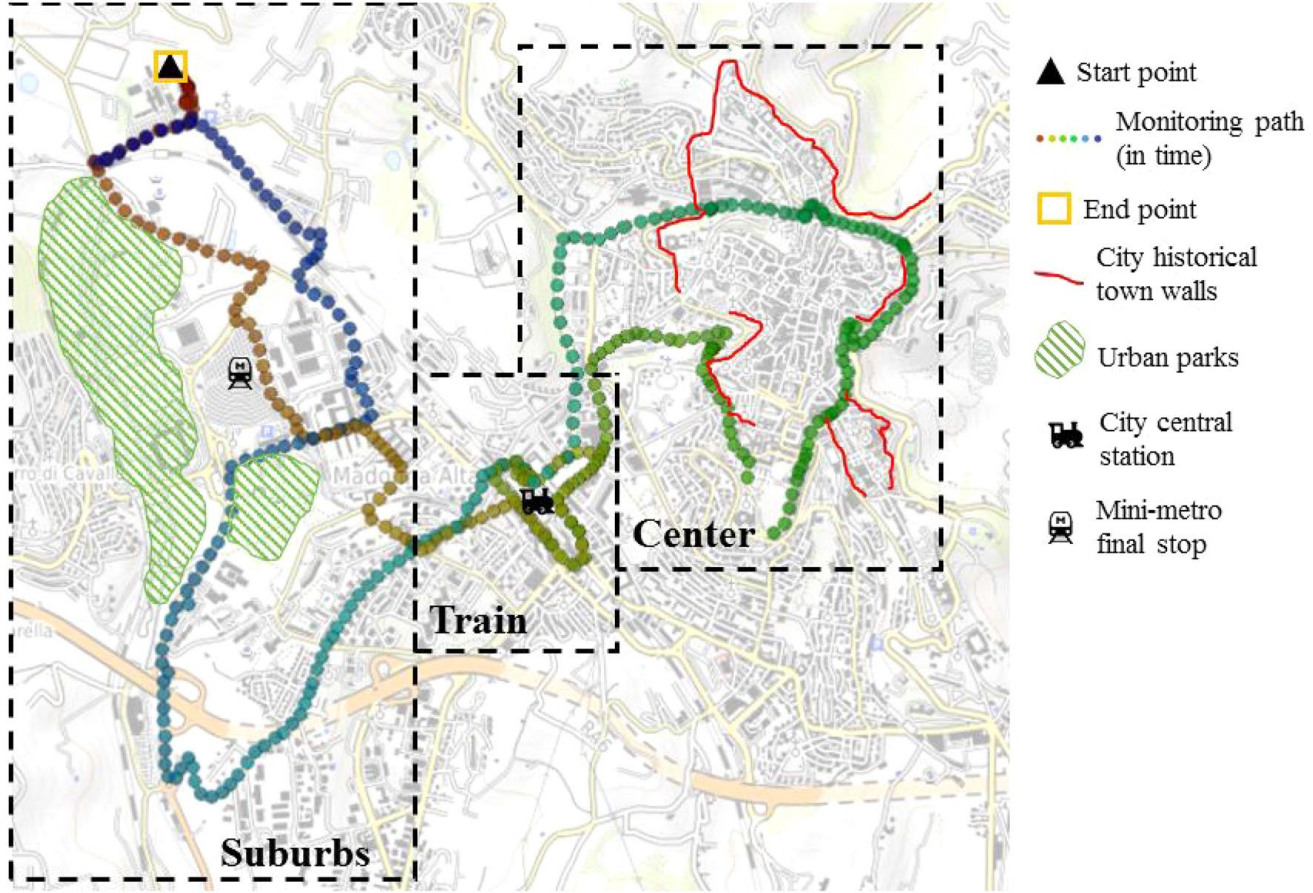
Pathway of monitoring campaigns, made via GPS Visualizer online application (https://www.gpsvisualizer.com/).

Both monitoring campaigns followed the very same pathway within the city of Perugia, in central Italy. According to the Köppen and Geiger classification, Perugia is classified as Cfa and is characterized by humid subtropical climate conditions^66^. The pathway (Fig. 2) is almost circular and ends where it starts. It is specifically planned to pass through and monitor areas characterized by different types of (1) urban morphology, (2) land-use, and (3) human activity. Under this scenario, three significantly different areas in a radius of almost 2 km from the main train station of the city were identified. These areas present different building densities, prevailing built materials, and amount of greenery and were already identified in previously published research of the same authors in Pigliautile et al.^67^. More specifically, the case study city of Perugia presents an urban structure that comprises: (1) a hilly Medieval city center which is densely built, mainly characterized by stones and bricks as prevailing built materials, and not fully accessible by vehicles; (2) modern urban neighborhoods developed in proximity of the main train station and the main infrastructures connecting the historical city to its surroundings, that are similarly characterized by mid-rise buildings but wider roads, a higher amount of pollutants and anthropogenic heat sources, and prevailing asphalt and concrete as built materials; (3) suburbs that are outer from the center, sparsely built, and with a higher amount of greenery. Further details of each area with respect to the monitoring path can be found in Table 4. Moreover, the monitoring path was devised so as to be accessible by the equipped van and to be completed in less than one hour in order to minimize the environmental data elapsed time-dependency and thus to focus on spatial variability^56^. However, due to vehicular traffic, the day-2 monitoring transect exceeded the one-hour duration by 10 min.

Concerning mobile monitoring, a vehicle’s speed of 10 km/h is recommended by Oke^36^, while according to Taha et al.^56^ measurements of air temperature at vehicle speed lower than 10 km/h should be discarded. Other mobile monitoring studies, however, reported vehicle speeds above 30 km/h^47,55,57,59^. Here, in order to maintain the lowest possible speed within the city area and get a substantial spatial resolution, vehicle speed was maintained around 20 km/h and hence measurements were taken approximately every 50 m.

**Table 4:** Clustered areas’ details.

Clustered area	Coverage in progressive distnace in m	Abbreviation
Suburbs	0–4600	Suburbs-1
Train	4600–7900	Train-1
Center	7900–14,100	Center
Train	14,100–15,300	Train-2
Suburbs	15,300-end	Suburbs-2

## Results and discussion

### Boundary conditions

**Figure 3:**
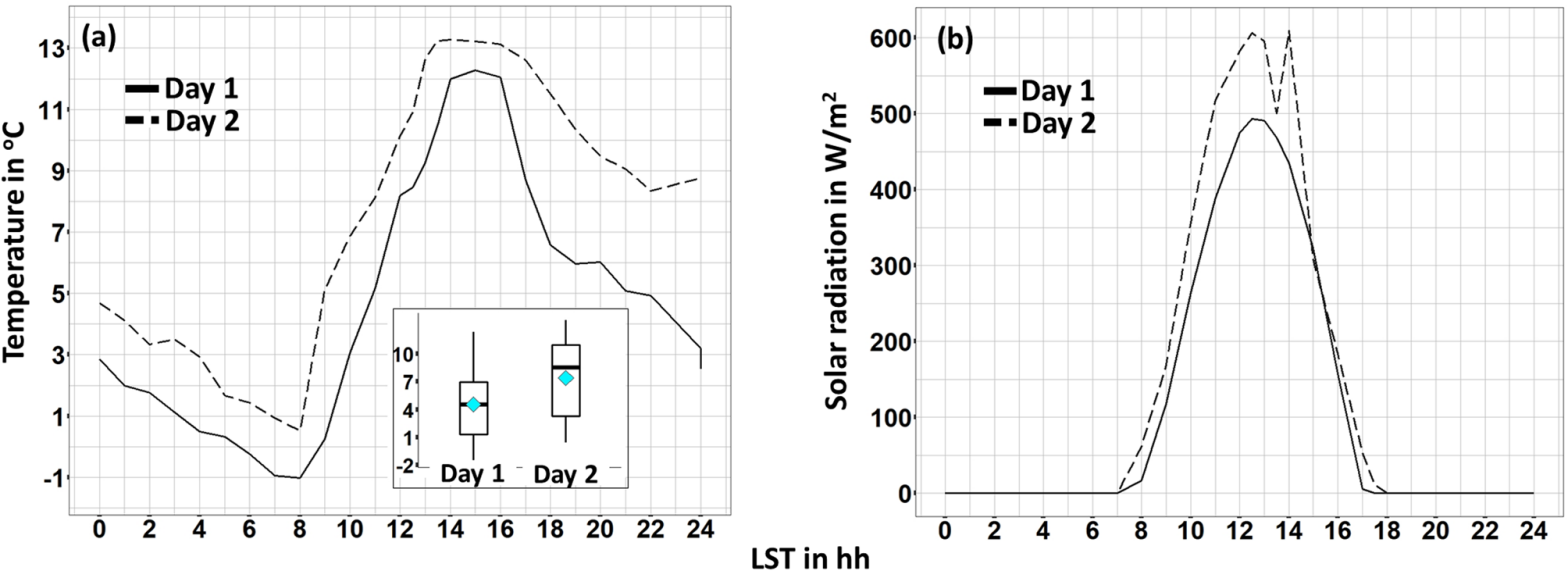
(**a**) 24 h air temperature profile for both day-1 and day-2, (**b**) 24 h solar global radiation profile for both day-1 and day-2.

At first, data derived from a stable weather station built on the roof of the University of Perugia^68^ were retrieved for defining the boundary conditions of each monitoring campaign in terms of air temperature and shortwave radiation. As it can be seen in Fig. 3, profiles of air temperature are rather similar for both days. The daily-mean air temperature values are 4.5 $^\circ \hbox {C}$ and 7.4 $^\circ \hbox {C}$ concerning day-1 and day-2, respectively. Furthermore, the mean air temperature values during the time-frame of the monitoring campaigns are 9.4$^\circ \hbox {C}$ and 9.3$^\circ \hbox {C}$ concerning day-1 and day-2, respectively, whilst the corresponding standard deviations are $0.4 ^{\circ }\hbox {C}$ and $0.5^\circ \hbox {C}$, respectively. Similarly, the daily-mean value of incoming shortwave radiation is $110\,\hbox {W/m}^2$ concerning day-1, whilst the corresponding maximum value is $492\,\hbox {W/m}^2$. The mean value of incoming shortwave radiation during the day-time monitoring campaign of day-1 is $485\,\hbox {W/m}^2$ and the corresponding standard deviation is $8\,\hbox {W/m}^2$.

### Intra-urban profiles of the microclimatic variables

**Figure 4:**
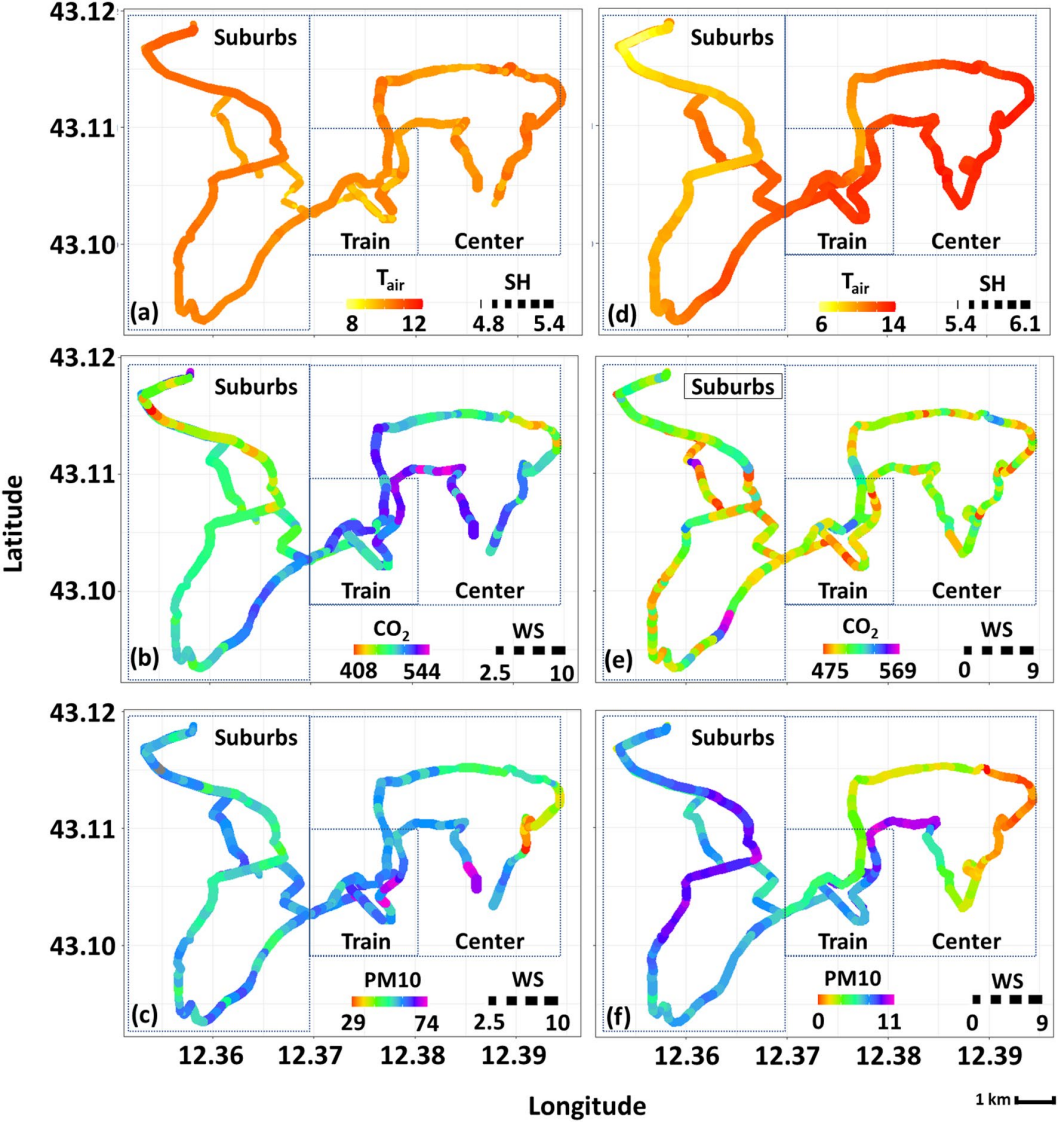
Day-time/Night-time monitoring. (**a**) Day 1—air temperature ($\hbox {T}_{\text {air}}$) versus specific humidity (SH), (**b**) day 1—$\hbox {CO}_2$ concentration versus wind speed (WS), (**c**) day 1—PM10 concentration versus wind speed (WS), (**d**) day 2—air temperature ($\hbox {T}_{\text {air}}$) versus specific humidity (SH), (**e**) day 2—$\hbox {CO}_2$ concentration versus wind speed (WS), (**f**) day 2—PM10 concentration versus wind speed (WS). $\hbox {T}_{\text {air}}$ is in $^{\circ }\hbox {C}$, SH is in $\hbox {g}_{\text {v}}/\hbox {kg}_{\text {a}}$, $\hbox {CO}_2$ and PM10 in ppm, and WS in m/s$^2$.

A representation of intra-urban variations of some of the collected variables can be seen in Fig. 4 where air temperature (images a and d), $\hbox {CO}_2$ (images b and e), and PM10 (images c and f) profiles across the followed monitoring paths are depicted with respect to day-1 and day-2. Moreover, further information is given by varying dot size with respect to the desired variable. Here, the size of each illustrated circle-point varies with respect to the corresponding specific humidity (SH) and wind speed (WS) values concerning the air temperature and air pollutant images, respectively. Through this representation, some elementary conclusions can be made, e.g. that high-temperature values occur simultaneously with high values of specific humidity. Also, localized hot-spots with respect to each variable can be identified. For instance, within the monitoring duration of day-1, the highest values of temperature were recorded on the peripheral area of the city center and within the suburbs-2 area. On the night hours of day-2, the higher air temperature values were recorded also in the area of the railway station and in the center. Similarly, the highest values of $\hbox {CO}_2$ and PM10 concentration were recorded mainly at both railway and its neighboring areas, especially on day-1. During the monitoring hours of day-2, the atmosphere within the investigated area was rather clear in terms of PM10 concentration.

**Figure 5:**
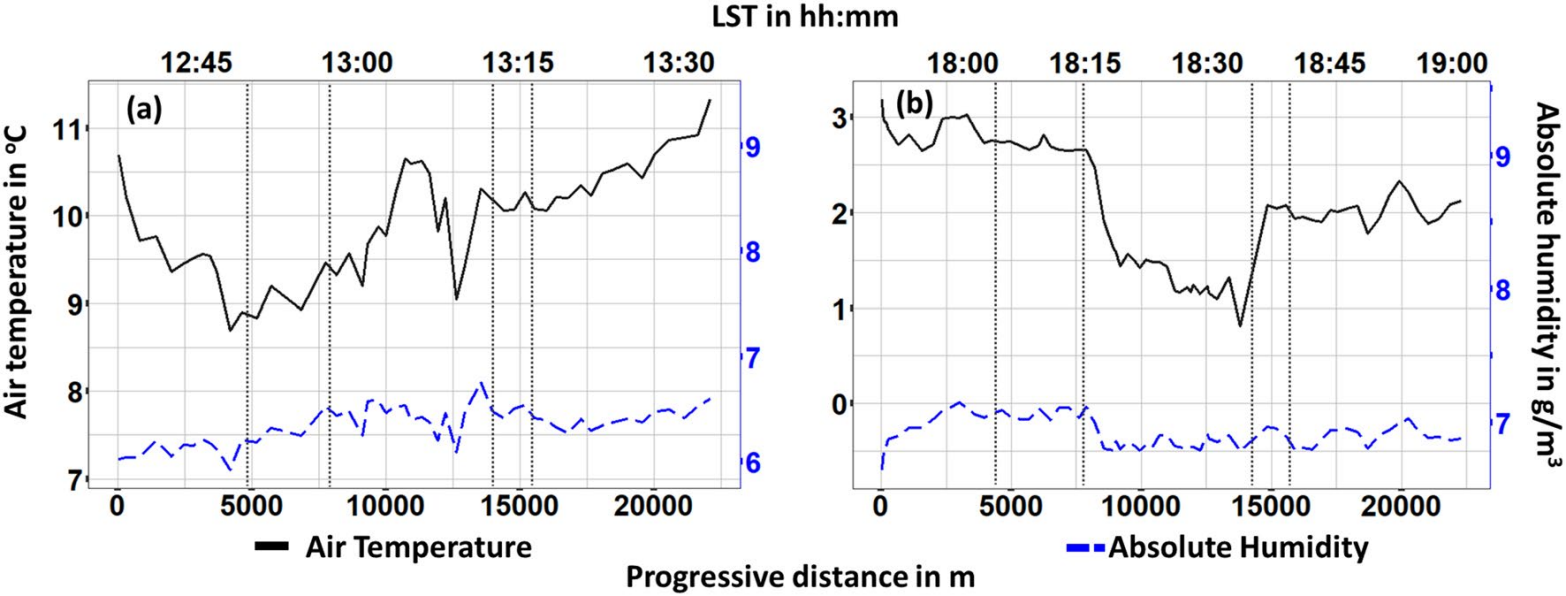
Air temperature and absolute humidity for (**a**) day 1, (**b**) day 2 monitoring. Vertical dotted lines stand for the boundaries in-between suburban (first and fifth section), train (second and forth section) and center area of the city.

A more precise picture of the variable profiles can be observed through the illustration of the corresponding time-series. In Fig. 5 the temperature profiles during the two monitoring campaigns are illustrated. A temperature gradient ($\Delta \hbox {T}_{max}\approx 1.5^{\circ }\hbox {C}$) can be seen between Train-1 and Center areas within day-1. An adverse but more steady gradient can be seen within day-2.

**Figure 6:**
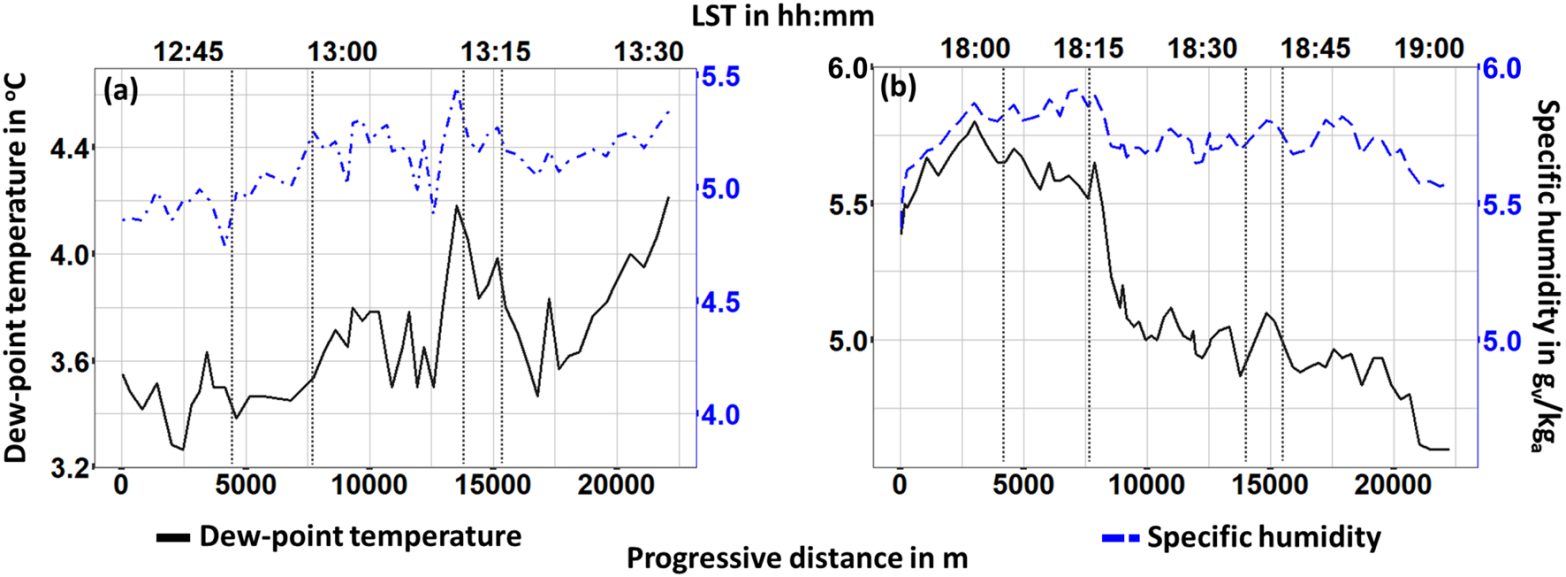
Dew-point temperature and relative humidity (**a**) day 1, (**b**) day 2 monitoring. Vertical dotted lines stand for the boundaries in-between suburban (first and fifth section), train (second and forth section) and center area of the city.

The temperature substantially dropped ($\Delta \hbox {T}_{max} \approx -1.5 ^{\circ }\hbox {C}$) while entering the Center area of the city and substantially increased when approaching Train-2 area ($\Delta \hbox {T}_{max} \approx 1 ^{\circ }\hbox {C}$). During both days, a steep drop and increase of temperature can be spotted within the last meters of the Center area. This trend is more evident within day-1 and is attributed to a substantial tree coverage within the specific street crossed in that area. Unlike relative humidity, absolute humidity, do not depend on temperature. However, here, a rather stable profile of absolute humidity can be observed during both day-1 (Standard deviation = $0.2\,\hbox {g/m}^3$) and day-2 (Standard deviation = $0.1\,\hbox {g/m}^3$), mainly due to the absence of water areas or large green areas. A similar profile is observed for specific humidity profile with standard deviation that also do not overpass $0.2\,\hbox {g}_{\text {v}}/\hbox {kg}_{\text {a}}$ and 0.1 $\hbox {g}_{\text {v}}/\hbox {kg}_{\text {a}}$ during day-1 and day-2, respectively (Fig. 6). Dewpoint temperature (DT) is an alternative way of capturing humidity and comfort and it is regarded as a more accurate metric since it is an absolute measurement. Moreover, it is also used to evaluate moisture, especially during spring and summer periods. During day-1 a significant gradient ($\Delta \hbox {D}\hbox {T}_{max} \approx 0.5 ^{\circ }\hbox {C}$) towards higher values can be seen as the station was moving from the Center area to the Train-2 one. A rather adverse profile is observed during day-2. Dewpoint temperature decreased as entering into Center area ($\Delta \hbox {D}\hbox {T}_{max} \approx -0.6 ^{\circ }\hbox {C}$) and remained almost stable ($\Delta \hbox {D}\hbox {T}_{max} \approx 4.8 ^{\circ }\hbox {C}$) up to the end of the campaign.

**Figure 7:**
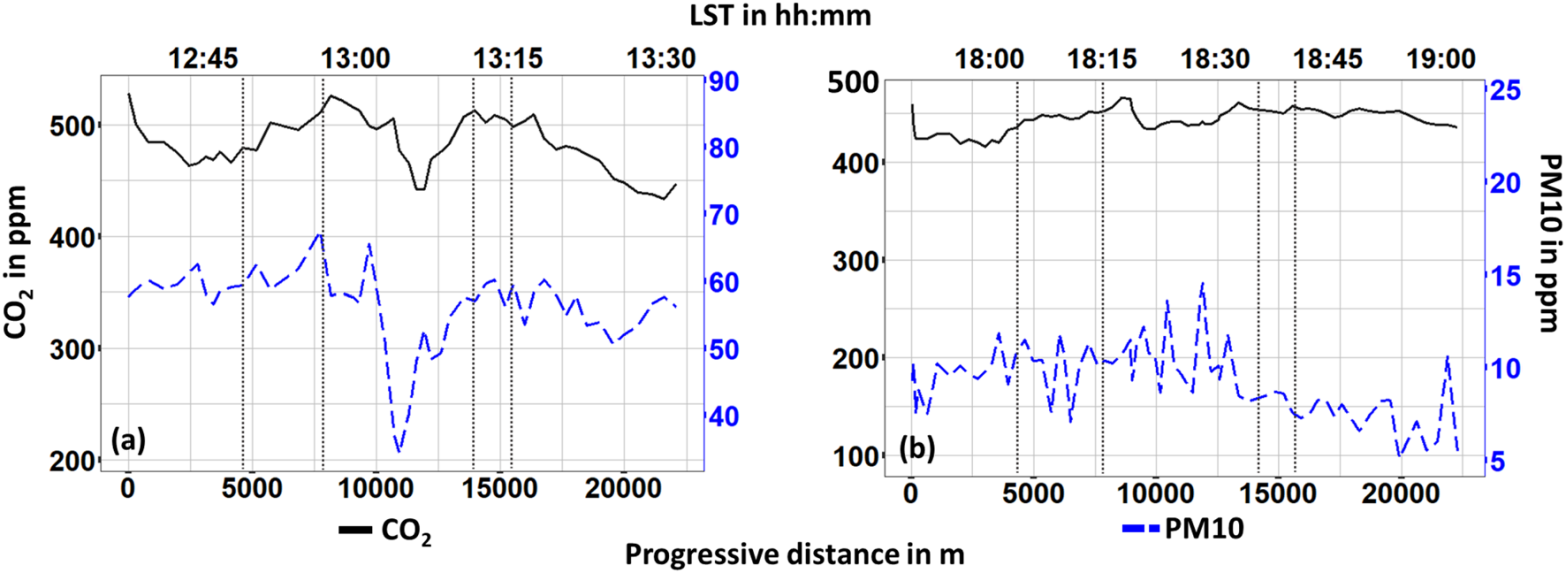
$\hbox {CO}_2$ and PM10 concentration (**a**) day 1, (**b**) day 2 monitoring. Vertical dotted lines stand for the boundaries in-between suburban (first and fifth section), train (second and forth section) and center area of the city.

Figure 7 shows the concentration levels of $\hbox {CO}_2$ and PM10, i.e. two key metrics of air pollution within an urban microclimate. Concerning $\hbox {CO}_2$, no substantial variations were recorded during both day-1 (Standard Deviation = 27.4 ppm) and day-2 (Standard Deviation = 12.3 ppm) time monitoring campaigns. However, on day-1, a small drop ($\Delta \hbox {CO}_{2max} \approx -70\,\hbox {ppm}$) of $\hbox {CO}_2$ concentration can be noticed within Center area. At the same day and point a small reduction ($\Delta \hbox {PM}10_{2max} \approx -30\,\hbox {ppm}$) can be observed also for PM10 concentration. This drop is likely due to the physical characteristics of the specific spot. It is an open-air spot and hence wind could locally remove pollutants. Also, a localized and short-term decrease in vehicular traffic might have occurred. Concerning PM10 during day-2, no significant variations were observed (Standard Deviation = 2.7 ppm).

**Figure 8:**
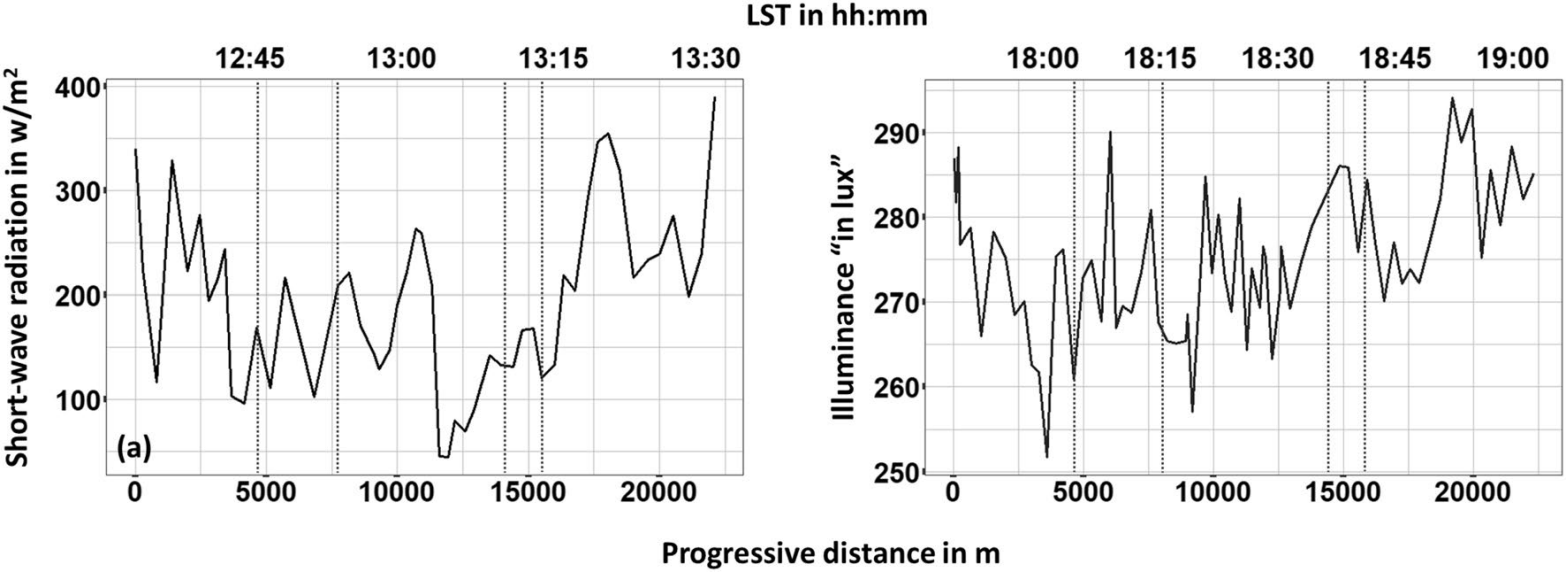
Solar-wave radiation and illuminance (**a**) day 1, (**b**) day 2 monitoring. Vertical dotted lines stand for the boundaries in-between suburban (first and fifth section), train (second and forth section) and center area of the city.

Short-wave radiation regulates urban microclimate during the day-time, whilst illuminance is a good indicator of anthropogenic action during the later hours of the day when sunlight is absent. Therefore, in Fig. 8 the profiles of shortwave radiation and illuminance are presented for day-1 and day-2, respectively. The results presented in this figure represent the average value of the data retrieved from the five sensors for both short-wave radiation and illuminance. Globally speaking, both solar radiation and illuminance are depending on the climatic zone of the investigated area, the time of the year, and the overall urban infrastructure. Of course, illuminance is a rather sensitive variable affected by various boundary conditions and hence its values significantly fluctuate around the mean value ($\hbox {E}_{vmax}$ = 347 lux and $\hbox {E}_{vmin}$ = 229 lux). Nevertheless, several peaks can be seen within Train-1, 2, and Center areas where the most anthropogenic activities take place (Fig. 8b). Similar fluctuations were found also concerning shortwave radiation (Fig. 8a). Overall, shortwave radiation follows a somehow similar profile with air temperature. For example, an increase ($\Delta \hbox {SR}_{max} \approx 100\,\hbox {w/m}^2$) can be seen as entering the Train-1 zone, while a steep drop up to $205\,\hbox {w/m}^2$ can be seen within the Center area when the station turned to a well-shaded street. The highest values were measured within the substantially unshaded areas of Suburbs-1 and 2.

**Figure 9:**
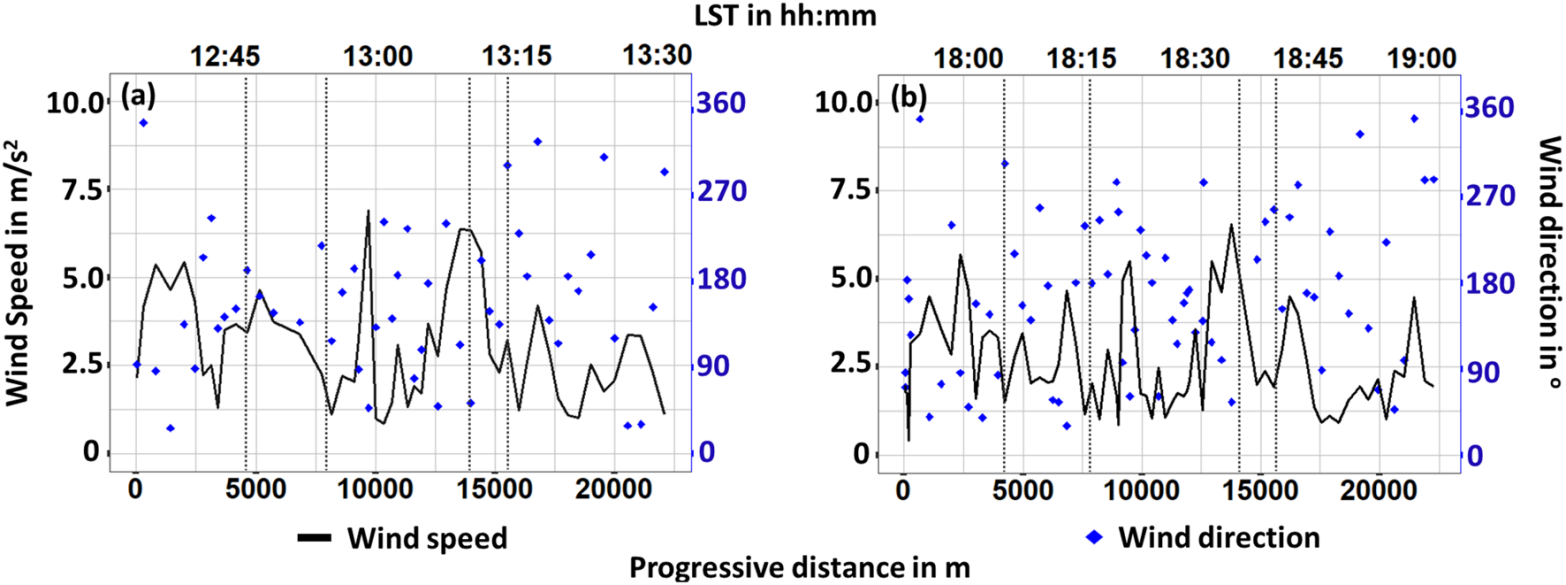
Wind speed and direction (**a**) day 1, (**b**) day 2 monitoring. Vertical dotted lines stand for the boundaries in-between suburban (first and fifth section), train (second and forth section) and center area of the city.

Day-1 and 2 were specifically chosen for the presented monitoring campaigns due to their relatively stable boundary conditions. As a result, wind speed deviations were rather small (Fig. 9), i.e. the wind speed standard deviations were $2.1\,\hbox {m/s}^2$ and $1.8\,\hbox {m/s}^2$ for day-1 and day-2 respectively. Two peaks can be observed as entering and leaving from the Center area owing to the corresponding open-air location, while inside the historic walls where the streets are substantially narrower wind speed was lower. Wind direction was in general towards North either North–East (0$^{\circ }$–90$^{\circ }$) or North–West (270$^{\circ }$–360$^{\circ }$). It should be noted that the highest values of wind speed occurred most of the time together with North–East wind.

**Figure 10:**
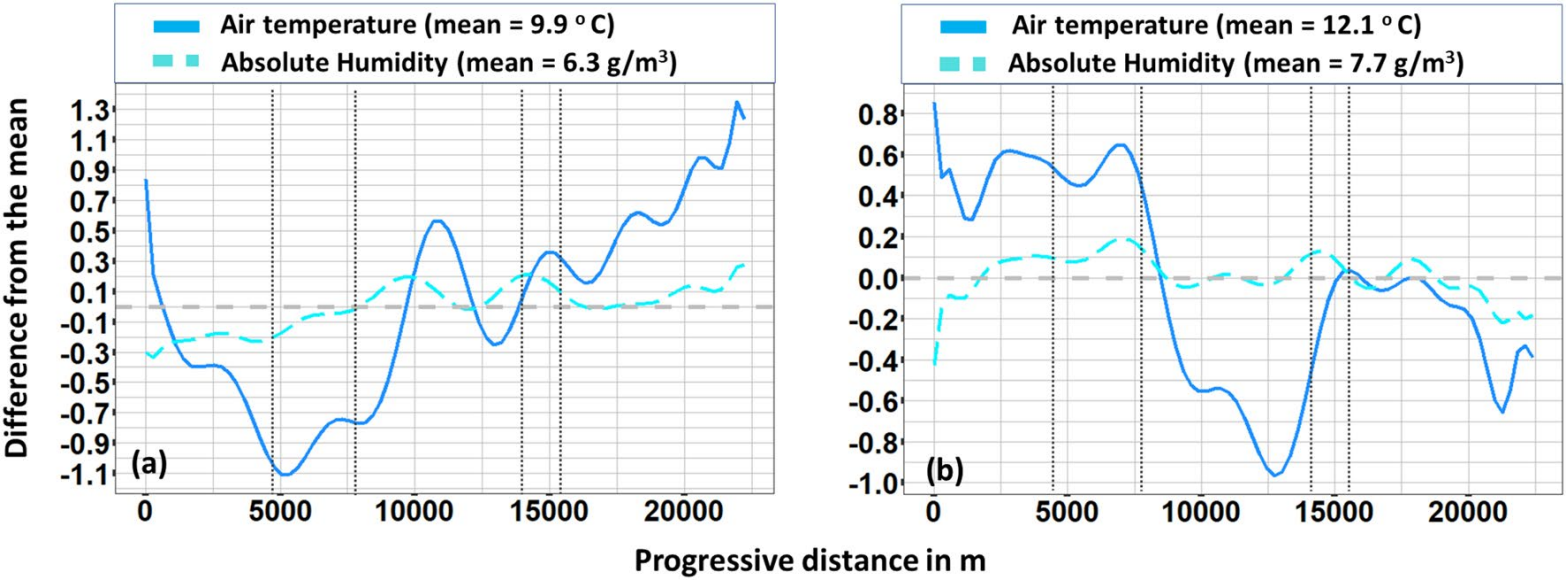
Deviations from the mean value. (**a**) day 1—air temperature and absolute humidity, (**b**) day 2—air temperature and absolute humidity. Vertical dotted lines stand for the boundaries in-between suburban (first and fifth section), train (second and forth section) and center area of the city.

Figure 10 presents the deviation of air temperature and absolute humidity with respect to the corresponding mean value. Significant deviations have been found concerning air temperature within both monitoring campaigns. For instance, during day-1 deviations ranged from − 1.1 to 1.3 $^{\circ }\hbox {C}$. The peak negative deviation from the mean value, i.e. 9.9 $^{\circ }\hbox {C}$, was recorded as approaching the Train-1 area while the first positive peak deviation was recorded within the Center area. Even higher positive peak deviation values were recorded within Suburbs-2 area owing to their unshaded and open-air environment. A rather adverse profile can be observed during day-2. The corresponding deviations ranged from − 1.0 to 0.8$^{\circ }\hbox {C}$. However, the global positive peak deviation was found for the Train-1 area while the global negative peak was found for the Center area. On the other hand, absolute humidity deviations as compared to the average value were found rather low.

**Figure 11:**
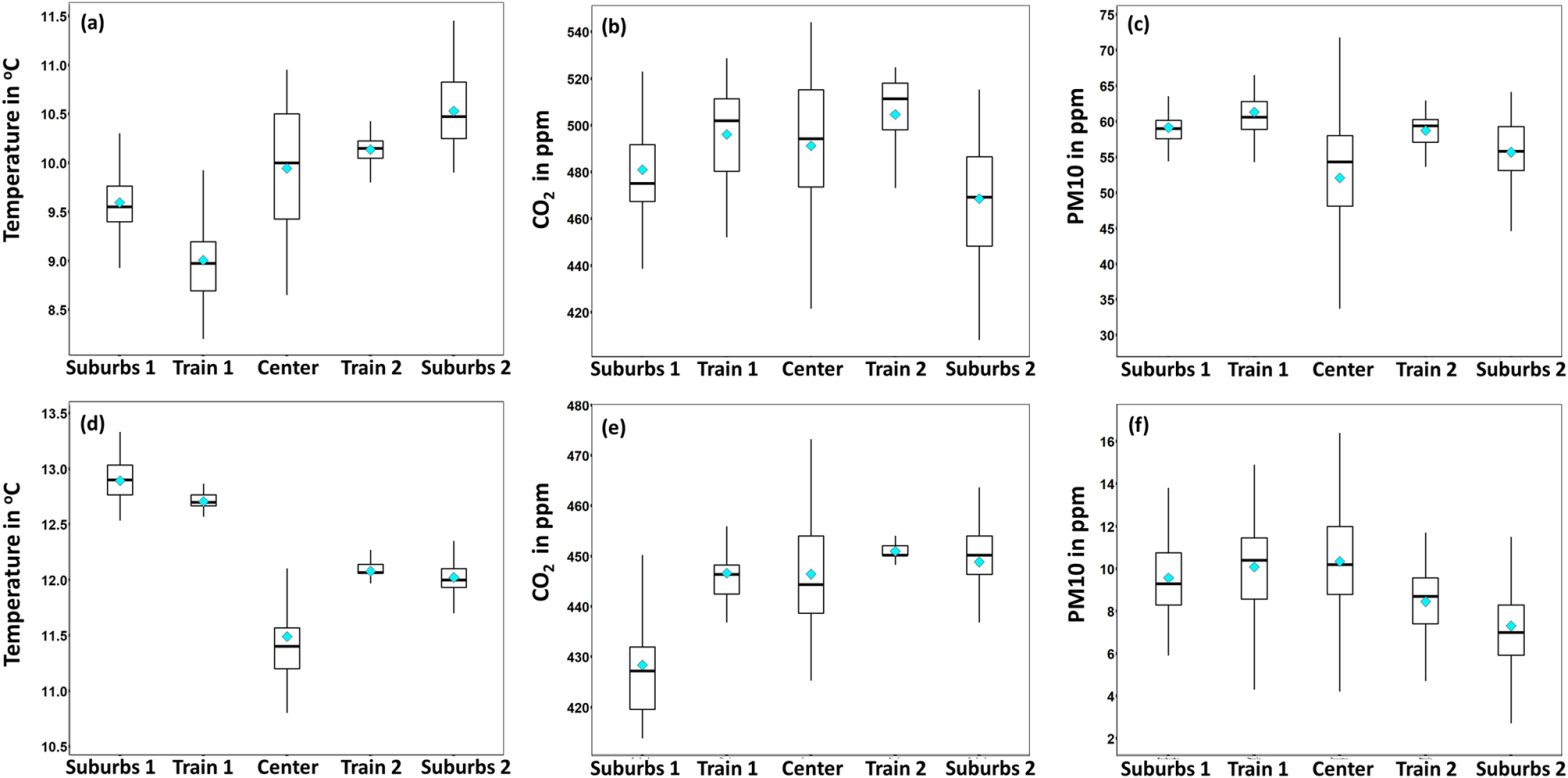
Cluster analysis of air temperature and air pollutants.

**Figure 12:**
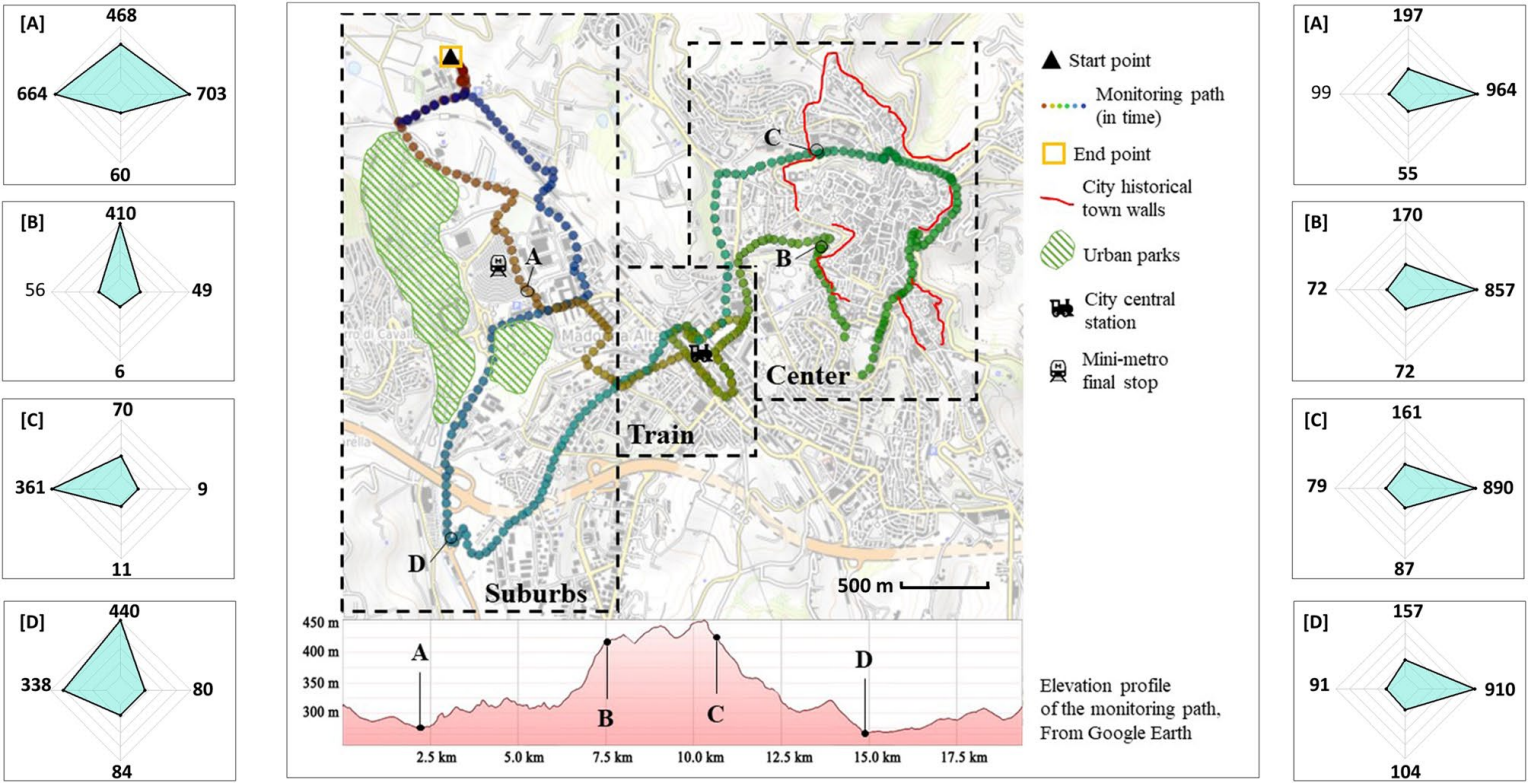
Directional representation of shortwave radiation ($\hbox {W/m}^2$—left column) and illuminance (lux—right column). The monitoring path map was made via GPS Visualizer online application (https://www.gpsvisualizer.com/).

In Fig. 11, a cluster analysis of the monitoring path can be seen with respect to different areas of the city. During day-1, the highest values of air temperature (Fig. 11a) were recorded within the suburbs 2 area. The air temperature was slightly lower within Train 2 and Center areas. However, concerning the latter area, the distribution was wider since this area comprises both narrow streets and open places. On the other hand, during day-2, the lowest air temperature values were recorded in Center area and the highest in the Suburbs 1 (Fig. 11b). Concerning $\hbox {CO}_2$, during day-1, the higher concentration values were recorded within the Train and Center areas (Fig. 11c), while during the day-2, except for Suburbs 1 area, all areas were found with rather similar concentration values (Fig. 11d). Concerning PM10, during day-1, the concentration within the Center area was found slightly lower as compared to the rest areas (Fig. 11e), while during day-2, a rather inverse profile was recorded Fig. 11f). In Fig. 12 the directional profiles of shortwave radiation (day-1) and illuminance (day-2) are presented at different spots within the monitoring path. As it can be seen, especially for the shortwave radiation, the direction of the incident radiation varies among different spots within the city, due to the varied urban morphology, e.g. open-air areas in the suburbs and narrow streets within the center area. On the other hand, illuminance levels during night-time transect did not vary substantially in terms of incident light direction but in terms of absolute value.

### Statistical analysis of the experimental data

#### Descriptive statistics

**Figure 13:**
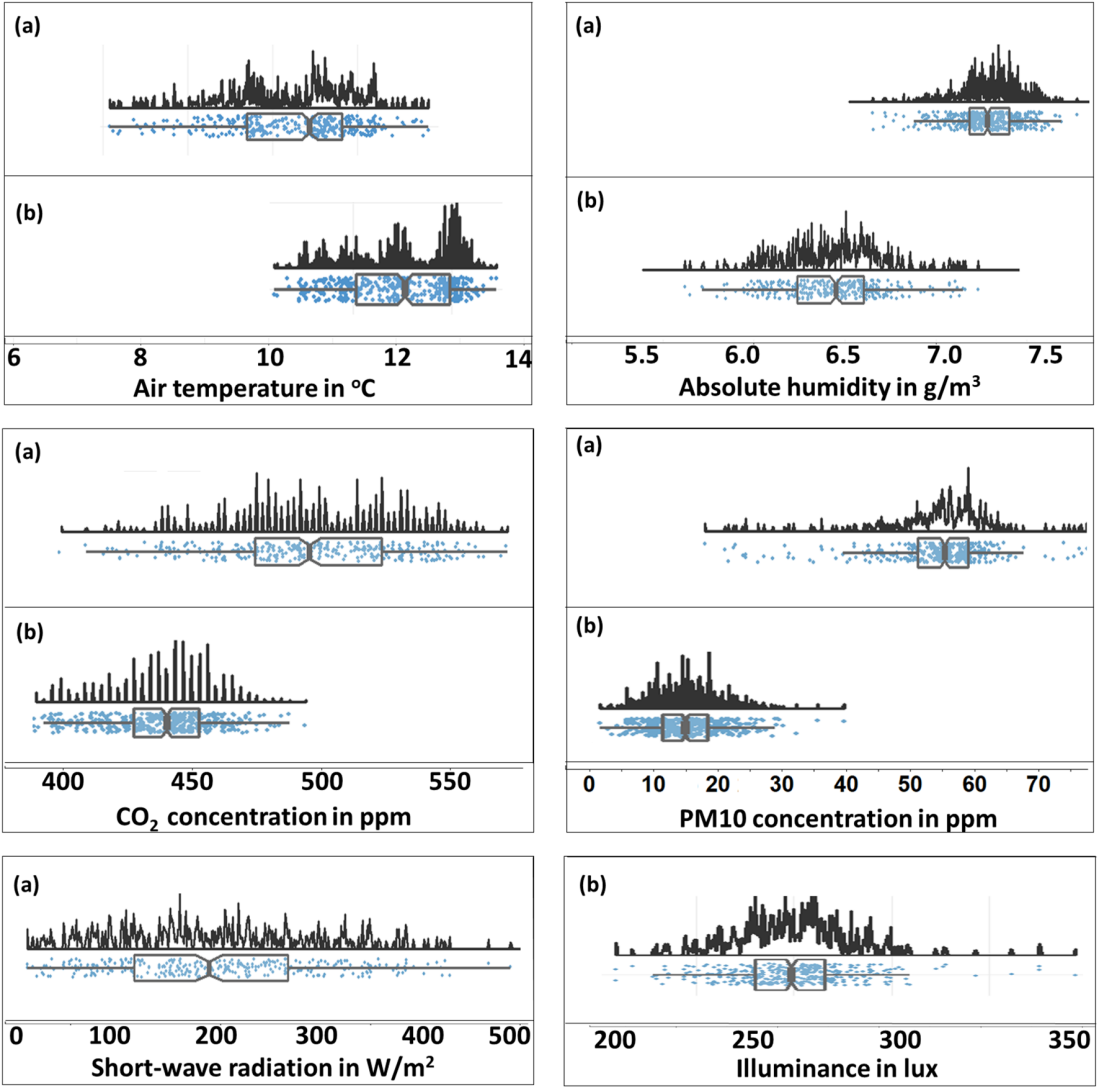
Probability density and boxplot of the measured variables within (**a**) day 1, (**b**) day 2.

Figure 13 illustrates the probability density and the central tendency of the monitored variables during the campaigns. Air temperature within day-1 is distributed approximately from 8 to 12 $^{\circ }\hbox {C}$ with a mean value equal to $9.9^{\circ }\hbox {C}$, while on day-2 air temperature is distributed from 10 to $13.4^{\circ }\hbox {C}$ with a mean value equal to $12.1^{\circ }\hbox {C}$. Temperature values were slightly higher during the night-time monitoring campaign due to the imminent ending of the winter period. On the contrary, absolute humidity values of day-2 were lower than the ones of day-1 with corresponding mean values equal to $6.9\,\hbox {g/m}^3$ and $6.3\,\hbox {g/m}^3$ respectively. The distributions of air pollutants, i.e. $\hbox {CO}_2$ and PM10, can be seen on Fig. 13. The widest distribution, as well as the highest values concerning both $\hbox {CO}_2$ and PM10, occurred on day-1, most likely due to higher vehicular traffic and other human-induced activities that take place more frequently during the daytime. The corresponding mean values are 484 ppm and 56 ppm concerning $\hbox {CO}_2$ and PM10 respectively. During day-2 $\hbox {CO}_2$ concentration is distributed within 413–477 ppm with a mean value equal to 443 ppm while PM10 concentration varies within 2–21 ppm with a mean value equal to 9 ppm. Shortwave radiation values during day-1 followed a rather wide distribution owing to urban morphology variations. In fact, shortwave radiation is distributed from 11 to $496\,\hbox {W/m}^2$ with a mean value equal to $196\,\hbox {W/m}^2$. A slightly narrower distribution is observed concerning illuminance during day-2, with values varying from 229 to 347 lux and a mean value equal to 274 lux.

#### Correlation analysis

**Figure 14:**
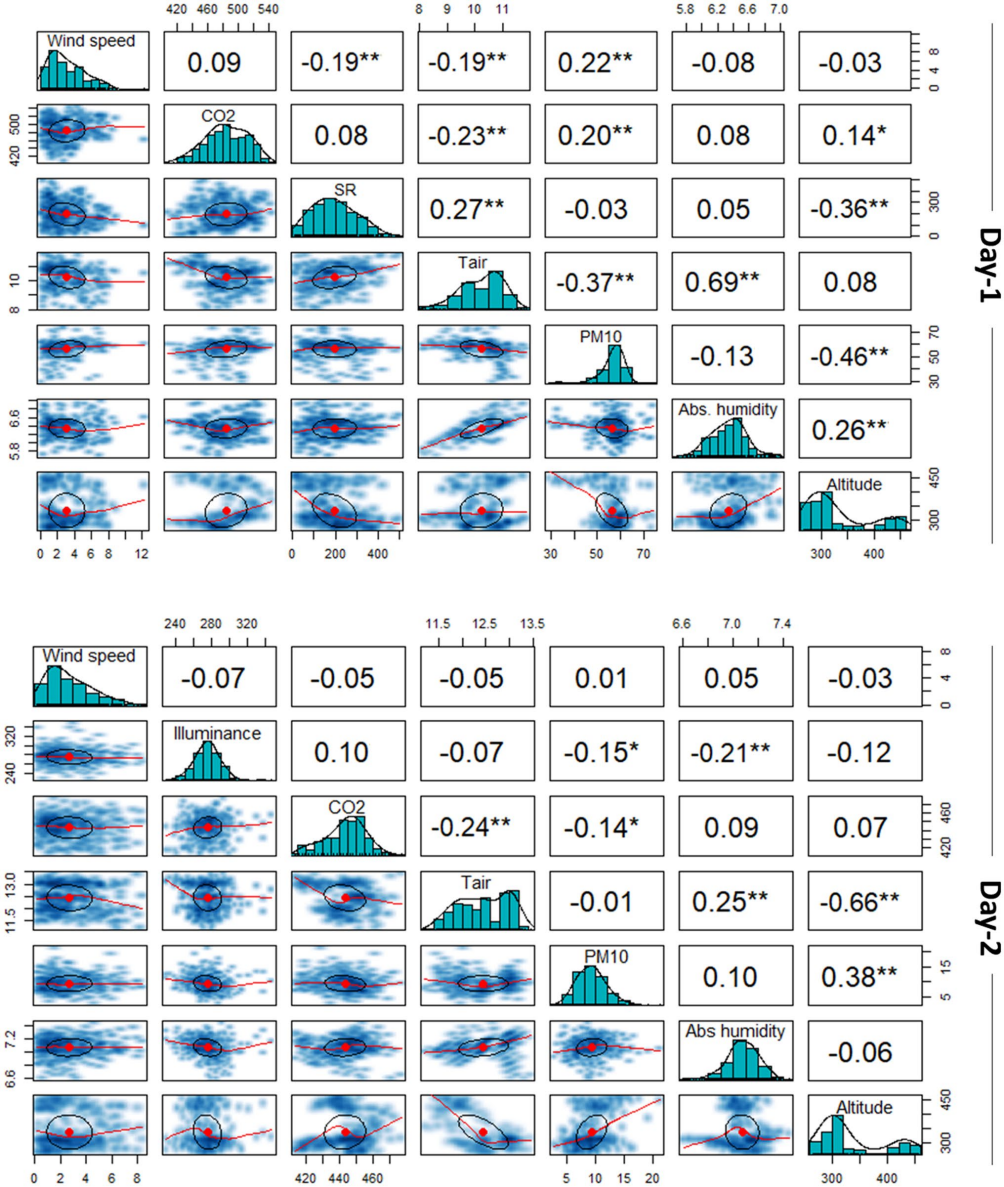
Correlation coefficients for day-1 and day-2. One star (‘*’) and two stars (‘**’) denote that the corresponding variable is significant at 5% and 1% level, respectively. Absence of star denotes no significant variable.

A Pearson’s correlation analysis, was performed for investigating possible primary linear relationships among the measured microclimate variables. The corresponding results are illustrated in Fig. 14. The diagonal of each matrix comprises variable histograms with kernel density estimations and the corresponding rug plots. On the part above the diagonal, the correlation coefficients are reported, whilst on the part below the diagonal the corresponding scatter plots with local regression (loess) fitted lines and covariance ellipses for displaying the strength of the relationship can be seen^69,70^.

During the day-time monitoring campaign, the most significant relationship was positive and found for air temperature and absolute humidity, i.e. r = .69, *p* < .001. Other moderate relationships were found for altitude and PM10 (r = .46, *p* < .001) and air temperature and PM10). Likewise, primary relationships within the measured variables were moderate to low during night-time monitoring. A negative primary relationship was observed concerning altitude and air temperature (r = − .66, *p* < .001) on day-2. In addition, during the same day, a positive relationship can be seen for altitude and PM10 (r = .38, *p* < .001).

#### Multiple linear regression analysis

**Figure 15:**
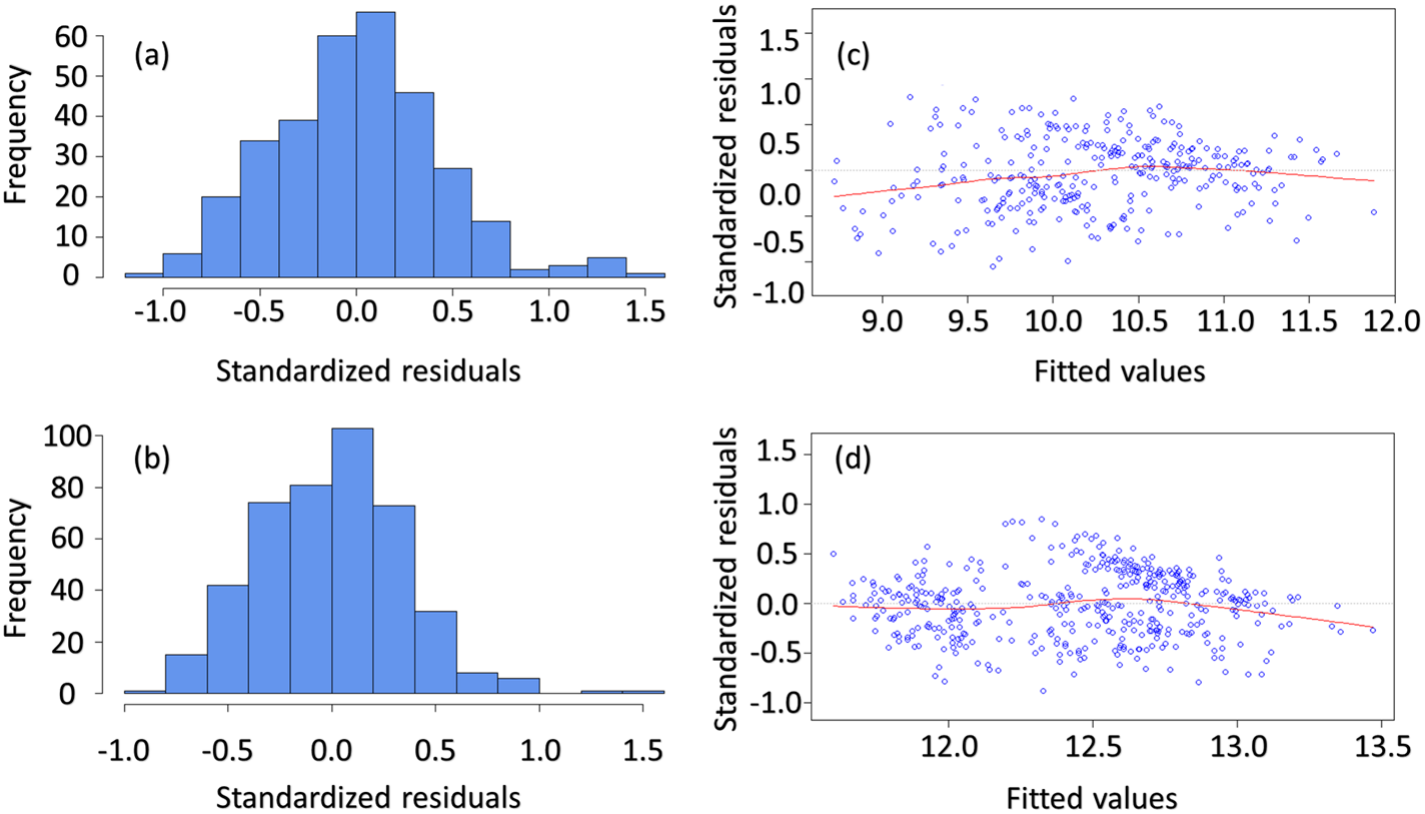
Residual analysis: (**a**) Histogram of frequency for day-1, (**b**) Residuals versus fits plot for day-1, (**c**) Histogram of frequency for day-2, (**d**) Residuals versus fits plot for day-2.

Multiple linear regression was employed to further investigate the relationship between air temperature, i.e. dependent variable, and the other measured microclimate parameters, i.e. independent explanatory variables. Air temperature is chosen as the dependent variable since is the main parameter that directly demonstrates the thermal environment of a typical urban environment. Moreover, datasets of both day-1 and day-2 meet the main assumptions of linear regression, i.e. multivariate normality, no multicollinearity, and homoscedasticity. The standardized residuals of the regression, i.e. the errors between observed and predicted values, are normally distributed (Fig. 15a,b). There is no evidence of significant multicollinearity since Variance Inflation Factors (VIF) of all explanatory variable are less than 4 and rather close to 1 (Tables 5 and 6) and the correlations among all independent variables have correlation coefficients less than .80 (Fig. 15). In addition, as it can be seen in Fig. 15c,d, the variance of the standardized residuals across the independent variables and the Loess-locally fit regression red-line that approximates zero, show now clear patterns across all levels of the independent variables.

Concerning both day-1 and day-2, the *p*-value of model’s F-statistic is < 2.2e−16, which is statistically significant, i.e. at least one explanatory variable is significantly related to the air temperature. Concerning day-1, a significant relationship with *p* < .001 is found between air temperature and each of absolute humidity, PM10, $\hbox {CO}_2$ and short-wave radiation (SR), whilst a significant relationship with *p* = .003 is found between air temperature and altitude (h). Concerning day-2, a significant relationship with *p* < .001 is found between air temperature and each of absolute humidity, PM10, $\hbox {CO}_2$, short-wave radiation (SR), and altitude (h), whilst a significant relationship with *p* = .001 is found between air temperature and wind speed (WS). The values of the adjusted R-squared suggest that the models explain a 68% and 58% of the variance of air temperature, concerning day-1 and day-2, respectively.

**Table 5:** Outcomes of the multiple linear regression—day-1.

					Confidence intervals		
	Estimate	Std. Error	t value	*p*-value	2.5 %	97.5 %	VIF
(Intercept)	0.661	0.807	0.819	0.413	− 0.926	2.248	–
SR	0.002	0.000	5.478	8.76e−08	0.001	0.002	1.336
h	− 0.002	0.001	− 3.007	0.003	− 0.003	− 0.001	1.823
$\hbox {CO}_2$	− 0.006	0.001	− 6.431	4.65e−10	− 0.008	− 0.004	1.182
PM10	− 0.032	0.004	− 7.089	8.84e−12	− 0.041	− 0.023	1.550
WS	− 0.009	0.012	− 0.726	0.468	− 0.033	0.015	1.095
AH	2.314	0.112	20.716	< 2e−16	2.094	2.534	1.010
F-statistic:	112.1						
*p*-value	< 2.2e−16						
Adjusted R-squared			0.679				

SR is Short-wave radiation, h is altitude, WS is wind speed and AH is absolute humidity.

**Table 6:** Outcomes of the multiple linear regression—day-2.

					Confidence Intervals		
	Estimate	Std. error	t value	*p*-value	2.5 %	97.5 %	VIF
(Intercept)	13.079	1.119	11.687	< 2e−16	10.879	15.279	–
h	− 0.007	0.000	− 21.098	< 2e−16	− 0.007	− 0.005	1.223
$\hbox {CO}_2$	− 0.007	0.001	− 5.163	3.73e−07	− 0.009	− 0.004	1.071
PM10	0.043	0.007	6.224	1.15e−09	0.029	0.056	1.239
WS	− 0.031	0.009	− 3.249	0.001	0.056	− 0.012	1.009
AH	0.735	0.129	5.679	2.50e−08	0.481	0.989	1.089
$\hbox {E}_v$	− 0.003	0.001	− 2.450	0.015	− 0.004	− 0.001	1.091
F-statistic:	97.89						
*p*-value	<2.2e−16						
Adjusted R-squared			0.577				

$\hbox {E}_v$ is illuminance, h is altitude, WS is wind speed and AH is absolute humidity.

## Conclusions

The current study reports on the application of advanced mobile monitoring techniques within a historical lively city of central Italy. Locating hot-spots with respect to each microclimate parameter, as well as identifying possible relationships among them is not trivial. Each city is characterized by its specific peculiarities. Perugia, the city chosen for the present study, is characterized by a diverse morphology. It comprises a city center with narrow and shaded streets with limited vehicular traffic, a more recent neighborhood developed around the main train station with substantial anthropogenic action, and several mostly residential suburban areas with open-air streets, and greenery. Here, a novel mobile monitoring station is implemented for monitoring the main micro-meteorological variables that affect climate and environment with high spatial granularity for both microscale, i.e. neighborhood-scale, and mesoscale, i.e. city-scale. The developed station can be easily adjusted to different type of vehicles such public transportation, electric cars or other dedicated monitoring vehicles. The main stimulus of its development was to gauge and map intra-urban deviations of the main variables determining urban microclimate, also imputable to anthropogenic actions. In this view, the scale of the analysis here reported is the neighborhood scale. Nevertheless, the system’s potentiality in retrieving analysis at higher-granularity is pointed out as well. Following an observational mobile-transect methodology, the station can access and monitor almost all areas accessible by car. Unlike mobile monitoring techniques implemented to date on a macro-scale within urban areas, the presented technique succeeds the detailed monitoring of scalar, vector, and directionally dependent variables. A start-up assessment was carried out in winter conditions, a period under-reported in particular in terms of mobile monitoring, and UHI studies. Results showed that determinants of urban microclimate and hence the quality of the urban environment can substantially vary within the very same urban context and with time (Table 7). Moreover, the direction of the incident shortwave radiation varied substantially among different spots of the monitoring path during the day-time transect. A directional dependency was found also for the illuminance levels during night-time transect. Overall, the outcomes of the study may represent a key missing piece for a state-of-the-art characterization of urban environmental quality. A more accurate discussion upon spatial accuracy achievable through the monitoring system according to its technical specifics and the data collection procedure is going to be provided in future research pushing forward a finer intra-urban microclimate variability description. Furthermore, future studies, aiming to extensively monitor and characterize specific urban environments, with respect also to temporal variations, should comprise a large number of transects during various hours of the day. Special focus should be given on the development of standards concerning elapsed time-correction of the data due to weather boundaries variation with respect to the duration of the transects, as well as on the seasonal comparison of parameters’ profiles and accurate calibration of the sensors prior to the monitoring campaigns. Further evaluation of urban environments under the framework of a wide monitoring network comprising also satellite and stable weather station data or other mobile stations, e.g. bicycle and wearable sensing techniques, can contribute towards effective data-driven decision-making policies with respect to risk and urban resilience planning.

**Table 7:** Maximum variations of measured variables across the monitoring path.

	Air Temperature ($^{\circ }C$)	Absolute humidity ($\hbox {g/m}^3)$	$\hbox {CO}_2$ concentration (ppm)	Pm10 concentration (ppm)	Solar radiation ($\hbox {W/m}^2$)	Illuminance (lux)	Wind speed ($\hbox {m/s}^2$)
Day-1	3.2	1.3	136	45	484.1	–	12.3
Day-2	2.3	0.8	64	19	–	118	8.3
